# Comparability of microbiota of swabbed and spit saliva

**DOI:** 10.1111/eos.12858

**Published:** 2022-02-26

**Authors:** Amke Marije Kaan, Bernd W Brandt, Mark J Buijs, Wim Crielaard, Bart JF Keijser, Egija Zaura

**Affiliations:** ^1^ Academic Centre for Dentistry Amsterdam Preventive Dentistry Amsterdam The Netherlands; ^2^ Netherlands Organisation for applied scientific research (TNO) Microbiology and Systems Biology Zeist The Netherlands

**Keywords:** 16S rDNA, microbiology, oral

## Abstract

In general, saliva is used for microbiota analysis in longitudinal studies, and several collection methods are being used. Using a robust sample collection procedure is important, as it may influence salivary composition. This study explored the comparability of the microbiota of swabbed and spit saliva. Twenty‐two females participated in this cross‐sectional study. The bacterial composition of the three saliva samples (swab collected by the participant (SW‐P), swab collected by the researcher (SW‐R), and spit (SP) was assessed by 16S rRNA gene amplicon sequencing. The bacterial composition of the swabbed and the spit saliva was significantly different irrespective of the operator, and Shannon diversity was significantly higher in spit saliva than in SW‐P and SW‐R. The salivary microbiota of spit and swabbed adult saliva differs significantly. Research on microbial composition therefore requires collection of similar saliva sample types in all study participants.

## INTRODUCTION

The oral cavity is a unique environment, harboring both soft tissues (e.g., buccal mucosa, gingiva, tongue), and non‐shedding, hard surfaces (teeth). Local factors of these different habitats, such as number and type of adhesive receptors, nutrient supply, oxygen levels, microbial competitors or collaborators, and local immune factors, influence colonization of micro‐organisms. These habitat‐specific environments lead to similarities between microbiota of the same niches between individuals [1].

The oral microbiota, the community of bacteria, fungi, viruses, archaea, and protozoa, play an important role in the development of oral diseases such as dental caries and periodontitis [2]. The interest in the role of oral microbes has grown, as besides oral diseases, a relationship between oral microbiota and systemic diseases, such as diabetes [3] and rheumatoid arthritis [4], has also been shown. In order to clarify the role of oral microbiota in systemic disease, longitudinal research is needed.

Robust sample collection methods are important in order to draw reliable conclusions based on research. Especially in longitudinal research involving infants, this requires attention, as the ability of young children to perform sample collection procedures changes with age. Variations in collection procedures may induce unwanted variation in microbial composition. As saliva is easy to collect and in contact with most surfaces, it is frequently used as a proxy sample of the oral microbiome [5]. Several methods for saliva collection are being used, such as passive drooling, oral rinse, pipetting and swabbing [6, 7, 8]. To date, little is known about the comparability of the microbiota of saliva collected in different ways.

In this short communication, we discuss some unexpected findings from a study comparing swabbed and spit saliva that may give useful insights for future studies using saliva for microbiota analysis.

## MATERIAL AND METHODS

This cross‐sectional study was approved by the Ethics Committee of the Academic Medical Centre, Amsterdam, The Netherlands (number NL66102.018.18, 16‐08‐2018) and was conducted in accordance with the declaration of Helsinki. Part of the data obtained was published previously [9]. We excluded females who did not master the Dutch language, who were younger than 18 years of age, and who had used antibiotics in the past 3 months. We included 22 females, who were instructed to refrain from oral hygiene practices 12 h before sample collection and to avoid drinking fluids other than water, as well as eating, smoking, or chewing gum at least 2 h before sampling. We aimed to collect samples in the morning in order to simplify adherence to these restrictions and to limit the effect of salivary compounds on microbial composition.

Two sample types were collected: a swabbed and a spit saliva sample. The swabbed sample was collected twice: first by the study participant herself and then by the researcher, using a COPAN eNAT (COPAN group) sample collection device consisting of a sterile swab and 1 ml eNAT buffer in a 10 ml tube. The swabbed sample was collected by keeping the swab in the lingual vestibule, below the tongue, for 20 s. Immediately after sample collection, the swab was placed in the tube containing buffer solution. Spit saliva was collected by asking the participant to swallow and then collect saliva that passively formed in the mouth into a 50 ml Falcon tube for a maximum of 2 min. The tube was weighed before and after saliva collection in order to calculate salivary flow rate. Each sample was put on ice immediately after collection and stored at −80 ⁰C within 2 h of sample collection. Detailed sample processing and 16S rRNA gene amplicon sequencing procedures have been described previously [9]. In brief, bacterial DNA concentration was determined by 16S ribosomal DNA quantitative polymerase chain reaction (qPCR) after DNA extraction and purification [10], after which the V4 region of the 16S rRNA gene was amplified [11] and sequenced (MiSeq; Illumina). The reads were denoised using UNOISE3 pipeline [12] and mapped into zero radius operational taxonomic units (zOTUs). HOMD version 14.51 [13] was used for taxonomic assignment of the zOTUs. The zOTU table was subsampled at an equal depth of 5000 reads per sample.

### Statistical analyses

The zOTU‐data was log‐2‐transformed for multivariate analyses of microbial profile data. PAST (PAlaeontological STatistics) version 3.20 [14] was used for Principal Component Analysis (PCA), PERmutational Multivariate ANalysis Of VAriance (PERMANOVA, using Bray‐Curtis distance and 9999 permutations) and calculation of Shannon diversity index. The Shannon diversity index was compared between related samples using Wilcoxon‐Signed‐Rank test in SPSS (IBM, version 25). For post‐hoc (pairwise) PERMANOVA, Bonferroni corrected *P*‐values were used. Results were deemed significant at *P* < 0.05.

The LEfSe biomarker discovery tool [15] was used to identify differentially abundant zOTUs by sample type. The all‐against‐all strategy and linear discriminant analysis (LDA) values of 3.0 and higher were additionally statistically tested with the Kruskall‐Wallis‐test. The Benjamini‐Hochberg procedure was used in R (version 4.0.2) to adjust for multiple testing and to control false discovery rate [16]. Adjusted *P*‐values were deemed significant at *P* < 0.05.

The Relative Abundance (RA) of genera was calculated and differences in abundance between sample types were statistically tested using Wilcoxon Signed rank test and Mann‐Whitney‐U tests. *P*‐values were deemed significant at *P* < 0.05.

### Assessment of shared taxa

For each sample type, the zOTUs with an abundance of at least 0.001% (five reads) in at least 25% of the participants were selected and visualized in a Venn diagram [17]. Next, the zOTUs were summarized per set in a total relative abundance, median, and range. Low abundant zOTUs were not excluded for RA calculations.

## RESULTS

Saliva samples were collected from 22 females (mean age ± SD: 33.1 year ± 5.3, ranging between 25 and 41 year) and salivary flow rate of the spit saliva was calculated (mean flow rate ± SD: 1.13 ml/min ± 0.52). In three participants, the duration of the spit saliva sample was shorter than 30 s, so no salivary flow rate was calculated.

**FIGURE 1:**
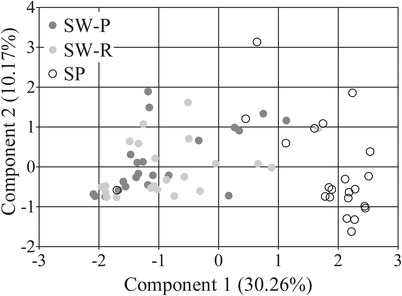
Principal Component Analysis plot of three saliva samples (SW‐P: swab collected by study participant; SW‐R: swab collected by researcher; SP: spit saliva)

**FIGURE 2:**
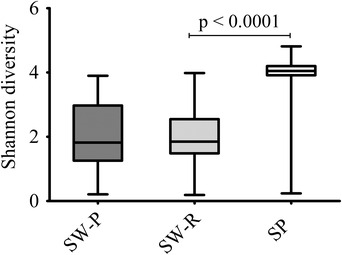
Comparison of Shannon diversity index of three saliva samples (SW‐P: swab collected by study participant; SW‐R: swab collected by researcher; SP: spit saliva)

**FIGURE 3:**
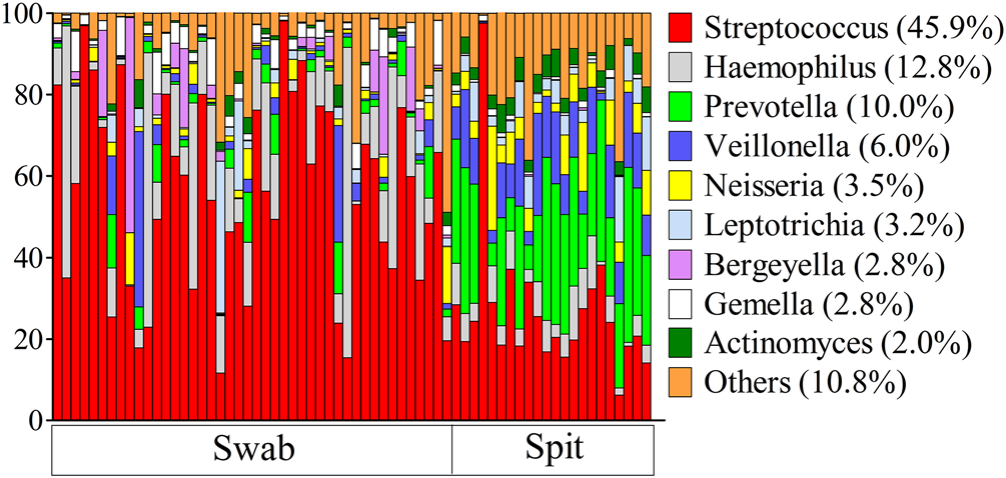
Most abundant genera per sample type

The bacterial composition of the three saliva samples (swab collected by the participant (SW‐P), swab collected by the researcher (SW‐R), and spit (SP)) was assessed. Interestingly, the bacterial composition of the swabbed and the spit saliva was significantly different irrespective of the operator (SW‐P: F = 11.52, *P* = 0.0003; SW‐R: F = 10.98, *P* = 0.0003) (Figure 1) and Shannon diversity was significantly higher (*P* < 0.001) in spit saliva (Figure 2). LEfSe identified no zOTUs that were differentially abundant between SW‐P and SW‐R, 41 zOTUs between SP (64.0% RA) and SW‐P (53.7% RA), and 57 between SP (75.6% RA) and SW‐R (61.0% RA) samples. The genera *Gemella* (*P* < 0.0001) and *Haemophilus* (*P* = 0.007) were significantly more abundant in swabbed saliva, while *Actinomyces, Veillonella, Prevotella*, and *Neisseria* (*P* < 0.0001) were significantly more abundant in spit saliva (Figure 3).

### Shared taxa between sample types

**FIGURE 4:**
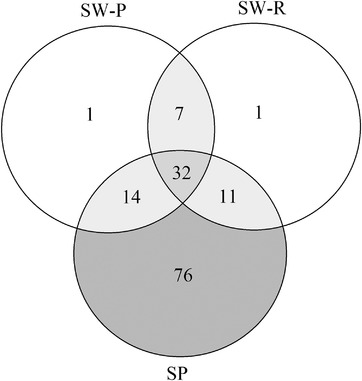
Venn diagram of the number of shared zOTUs of three saliva samples (SW‐P: swab collected by study participant; SW‐R: swab collected by researcher; SP: spit saliva)

The number of shared zOTUs and unique zOTUs per sample type were assessed (Figure 4). A total of 32 zOTUs were present in all three sample types in at least 25% of the study participants. Interestingly, only one zOTU was uniquely present in the two swabbed saliva samples, while 76 zOTUs were only present in spit saliva. Most of these 76 zOTUs belonged to the genera *Prevotella* (10.1% abundance), *Actinomyces* (1.9% abundance), or *Veillonella* (1.6% abundance).

## DISCUSSION

In this study, swabbed saliva showed a significantly different bacterial composition and a lower diversity in comparison to spit saliva. These results indicate that swabbed saliva is not a valid alternative for spit saliva for oral microbiota analysis.

The distinct microbial composition between swabbed and spit saliva found in this study may be a result of the removal of bacteria from oral mucosa on saliva collection using a swab. This can be seen in our results: swabbed saliva contained more significant zOTUs that are commonly related to mucosa (*Haemophilus)* [18] compared to spit, while spit saliva contained more significant zOTUs that are common inhabitants of dental plaque and subgingival surfaces (*Veillonella, Prevotella)* than swabbed saliva [18]. The latter suggests an active dislodgement of plaque bacteria from the dental surfaces into saliva. Also, the spit sample was significantly more diverse compared to the swabbed samples, suggesting tooth‐related bacteria were underrepresented in the swabbed sample. As the submandibular and sublingual glands empty saliva into the oral cavity via the sublingual caruncle, a possible explanation for the high diversity of spit saliva could be that the spit saliva contains micro‐organisms from multiple intra‐oral surfaces, while the swabbed saliva mainly contains micro‐organisms from the sublingual mucosa. Therefore, the spit sample may give a more complete representation of the oral microbiome compared to swabbed saliva.

Another possible explanation for the differences between spit and swabbed saliva may lie in differences in saliva stimulation upon sample collection. Passive drooling is a common procedure for saliva collection; however, it is difficult to maintain unstimulated collection as passive drooling might make study participants feel uncomfortable. In our study, the samples were mixed samples of unstimulated and stimulated saliva, as salivary flow can be stimulated upon swabbing (swabbed sample) and spitting (spit sample) [19]. A previous study comparing which type of sample patients preferred to collect – saliva by passive drooling, stool, or blood – showed that patients preferred saliva donation over stool and blood, but this preference declined after actual sample collection [20]. In that study, unfortunately, the sample collection procedure was not evaluated so it is unknown if passive drooling was maintained. This is especially important in saliva collection, since the comparability of bacterial profiles of stimulated and unstimulated saliva has been studied before and the results of these studies are conflicting [21, 22]. It is very important to report the exact way of saliva collection and the instructions given to the study participants, to be able to compare different studies.

In our study, transportation on ice and storage of all samples at −80 degrees Celsius within 2 h of collection was used to limit DNA degradation and bacterial growth. Also, a buffer for sample preservation was used for the swabbed samples, but not for the spit samples, which could have influenced our results. The buffer used in our study is a lysis buffer containing guanidine thiocyanate, which immediately lyses the cells to protect the bacterial DNA from degrading, and to inhibit bacterial growth [23]. Comparison of stool samples stored in −80 degrees Celsius and samples stored at room temperature in a buffer containing guanidine thiocyanate showed no significant differences on taxonomic level [24], so we expect the effect of this buffer on microbial composition to be limited. In theory, it could be expected that microbial diversity decreases in samples to which no buffer solution was added. Although no buffer was added to the spit saliva in this study, the diversity was significantly higher than seen for SW‐R and SW‐P. If a lysis buffer had been added, we would expect this difference to be even larger. Still, the influence of buffer solution on microbial composition is not entirely clear and requires more research.

In general, microbiota can be influenced by several factors, for example, antibiotics [25], thus we selected participants who did not use antibiotics. In saliva research specifically, the time of saliva collection is important, as its flow and some of its components, such as cytokines, are subject to diurnal fluctuations [26]. In order to limit the effect of different salivary components, we aimed to collect samples in the morning. As three participants were unavailable in the morning, samples from these participants were collected in the afternoon around 14 PM. We found no differences between samples that were collected at different time points.

Passive drooling is a common procedure for saliva collection in adults. However, it is not a suitable collection method for infants or young children, as they are unable to follow instructions or to spit saliva. Therefore, in several studies, saliva from infants was collected using a swab while saliva from a caretaker was collected by drooling [27, 28]. Our findings question the validity of addressing and treating spit and swabbed saliva as if they represent the same conditions. We therefore advise future studies to collect saliva from adults and infants in the identical way. This is especially important in longitudinal research, as switching to a different saliva collection method during the study may lead to bias.

To conclude, the salivary microbiota of spit and swabbed adult saliva differs significantly. Research on microbial composition therefore requires collection of similar saliva sample types in all study participants. This is especially important in longitudinal research and studies involving children.

## AUTHOR CONTRIBUTIONS

**Conceptualization**: AM Kaan, W. Crielaard, BJF Keijser, E. Zaura; **Investigation**: AM Kaan; **Methodology**: BW Brandt, MJ Buijs, E. Zaura; **Data curation**: BW Brandt; **Formal analysis**: AM Kaan, BW Brandt; **Writing‐ original draft**: AM Kaan; **Writing ‐ review and editing**: BW Brandt, MJ Buijs, W. Crielaard, BJF Keijser, E. Zaura; **Validation**: MJ Buijs; **Visualization**: AM Kaan; **Supervision**: W. Crielaard, E. Zaura

## CONFLICT OF INTEREST

No conflicts of interest.
